# Effects of a curriculum integrating critical thinking on medical students’ critical thinking ability in Iran: a quasi-experimental study

**DOI:** 10.3352/jeehp.2021.18.14

**Published:** 2021-07-05

**Authors:** Akbar Soltani, Mahboobeh Khabaz Mafinejad, Maryam Tajik, Hamideh Moosapour, Taha Bayat, Fatemeh Mohseni

**Affiliations:** 1 Endocrinology and Metabolism Research Center, Endocrinology and Metabolism Clinical Sciences Institute, Evidence Based Medicine Office, College of Medicine, Tehran University of Medical Sciences, Tehran, Iran; 2 Education Development Center (EDC), Department of Medical Education, Health Professions Education Research Center, Tehran University of Medical Sciences, Tehran, Iran; 3 Students’ Scientific Research Center, Tehran University of Medical Sciences, Tehran, Iran; 4 Department of Anesthesiology, Nursing School, Gerash University of Medical Sciences, Gerash, Iran; Hallym University, Korea

**Keywords:** Medical student, Thinking, Curriculum, Educational measurement, Iran

## Abstract

**Purpose:**

Improving physicians’ critical thinking abilities could have meaningful impacts on various aspects of routine medical practice, such as choosing treatment plans, making an accurate diagnosis, and reducing medical errors. The present study aimed to measure the effects of a curriculum integrating critical thinking on medical students’ skills at Tehran University of Medical Sciences, Iran.

**Methods:**

A 1-group pre-test, post-test quasi-experimental design was used to assess medical students’ critical thinking abilities as they progressed from the first week of medical school to middle of the third year of the undergraduate medical curriculum. Fifty-six participants completed the California Critical Thinking Skills Test twice from 2016 to 2019.

**Results:**

Medical students were asked to complete the California Critical Thinking Skills Test the week before their first educational session. The post-test was conducted 6 weeks after the 2 and half-year program. Out of 91 medical students with a mean age of 20±2.8 years who initially participated in the study, 56 completed both the pre- and post-tests. The response rate of this study was 61.5%. The analysis subscale showed the largest change. Significant changes were found in the analysis (P=0.03), evaluation (P=0.04), and inductive reasoning (P<0.0001) subscales, but not in the inference (P=0.28), and deductive reasoning (P=0.42) subscales. There was no significant difference according to gender (P=0.77).

**Conclusion:**

The findings of this study show that a critical thinking program had a substantial effect on medical students’ analysis, inductive reasoning, and evaluation skills, but negligible effects on their inference and deductive reasoning scores.

## Introduction

### Background

Critical thinking is described as the ability to pose a discriminating question in order to search for better ideas or to find better solutions [1]. The critical thinking process consists of collecting appropriate information, precisely evaluating the information, and using it to come to a considered conclusion. Since healthcare involves inherent uncertainties and is prone to diagnostic and management errors, improving physicians’ critical thinking could have a substantial effect on aspects of routine medical practice, such as choosing treatment plans, making an accurate diagnosis, and reducing medical errors [2]. Furthermore, critical thinking ability is strongly correlated with clinical competence and academic success [3]. Evidence shows that clinical clerkships and other forms of clinical experience provide the opportunity to enhance critical thinking through observation of more senior clinicians and gaining experience, but the impact of such experiences is insufficient [4]. As a result, medical schools are placing a major priority on assessing critical thinking, improving this ability using specialized teaching techniques, and providing sufficient educational opportunities [5]. The inadequacy of these methods has led to curricular reforms in an attempt to add critical thinking to all levels of education. Medical school instructors should be well versed in models of argument and should regularly encourage their students to engage in discussions during daily rounds, morbidity and mortality conferences, and any other teaching sites.

Despite the undeniable role of critical thinking ability in medical education, it is challenging to choose the best approach to enhance critical thinking skills. Several approaches to strengthen critical thinking skills have been developed. Investigators’ approach toward education focusing on critical thinking skills has generally reflected a viewpoint of teaching these skills as a general educational subject or as a subject integrated with specific knowledge. Students are most likely to benefit from learning critical thinking skills when they are delivered through specific educational modules within their curriculum [6]. However, the reported results in the literature are inconsistent; some researchers have found that education led to increases or decreases in critical thinking skills, while others have found that critical thinking was unchanged by a training program; therefore, it remains unclear how effectively critical thinking is being taught in medical schools. Instead of comparing medical students’ critical thinking before and after a short course, we analyzed changes in students’ ability across a 2. 5-year timeframe.

### Objectives

This study aimed to evaluate the effects of implementing a curriculum integrating critical thinking on medical students’ skills. The research questions were as follows: first, is there a significant change in the medical students’ critical thinking abilities as they progress from the first week of medical school to the third year of the undergraduate medical curriculum?; second, which critical thinking skills improve as students progress through the curriculum; and third, are there differences according to gender in critical thinking before and after the program?

## Methods

### Ethics statement

The institutional review board of Tehran University of Medical Sciences (TUMS) approved this study (IR.TUMS.REC 1390-16263). Informed consent was obtained from participants.

### Study design

This was a 1-group pre- and post-test quasi-experimental study.

### Setting

The study was carried out at TUMS. In 2006, TUMS commenced the development and implementation of a newly revised curriculum for delivering undergraduate medical education. A main feature of the revised curriculum is that it focuses more attention on the integration of critical thinking programs for training and assessing medical students. We assessed medical students’ critical thinking skills as they progressed from the first week of medical school to the third year of the preclinical phase in an undergraduate medical curriculum. A pre- and post-test method was used to assess the impact of the curriculum integrating critical thinking on medical students’ critical thinking skills. Participating students were asked to complete the California Critical Thinking Skills Test (CCTST; form B) the week before their first educational session. Post-test data were collected 6 weeks after the 2 and half-year program.

### Participants

All 91 medical students, who comprised 43 men (47.3%) and 48 women (52.7%) with an average age of 20±2.8 years, enrolled in the medical undergraduate curriculum at TUMS were invited to participate in the study. Sampling was done by the census method with the participation of all students enrolled in the medical undergraduate curriculum. The researchers included only data from students who completed both the pre- and post-intervention questionnaires (n=56), and excluded data from those who chose not to complete both tests. The valid response rate was 61.5%. The primary reason why some medical students did not complete the CCTST (form B) at both pre- and post-tests was absenteeism on the day of survey administration.

### Measurement tool

Demographic information, including age and gender, was collected in the first part of the questionnaire. The second part assessed the critical thinking skills of medical students using the valid and reliable CCTST form B, the Persian version of which contains 5 subscales: analysis, inference, evaluation, deductive reasoning, and inductive reasoning [7]. The CCTST contains 34 multiple-choice questions with a correct answer (with scores of 0–1). The maximum scores in the 5 sections are 9 for analysis, 11 for inference, 16 for deductive reasoning, 14 for inductive reasoning, and 14 for evaluation (Supplement 2). The scope of questions encompasses semantic analysis of a single sentence to a more complex combination of critical thinking skills. In other words, answering some of the questions of the CCTST requires extracting the correct inference from a series of assumptions, evaluating the options, and providing a reasoned justification for a conclusion. The total CCTST score ranges from 0 to 34, with higher scores reflecting stronger critical thinking skills. It took students about 45 minutes to complete the test. The reliability and validity of the CCTST assessment have been reported in previous publications [7]. In a study by Khalili and Hossein Zadeh [8], the reliability coefficient of the test, determined in terms of internal consistency and using the Kuder-Richardson Formula 20, was 0.62. The reliability coefficient of the subscales after factor analysis was in the range of 0.62–0.67. In the present study, the content validity of the Persian version of the questionnaire was confirmed by 8 faculty members in the fields of cognitive psychology, philosophy, medicine, and medical education. Its reliability was calculated using the Cronbach α coefficient, which was equal to 0.83. A researcher collected data before and after the courses over a 2 and half-year curriculum. The initial survey was conducted at the beginning of the first semester. The post-test was implemented 6 weeks after the 2 and half-year program.

### Interventional program

In this study, medical students participated in a critical thinking program. The experiment consisted of a critical thinking program implemented as part of the undergraduate medical curriculum of TUMS. The program consisted of 21 hands-on critical thinking skills lessons geared toward medical students in the basic sciences phase. A faculty member with expertise in the field of critical thinking ran the educational sessions, including 46 hours of training. To assist in the implementation of the intervention, at the first step a curriculum was designed consisting of a range of activities involving teaching critical thinking skills. Before pilot testing, the critical thinking program was reviewed by a team of 8 experts including a medical education specialist, a medical school instructor, a cognitive psychologist, and a medical practitioner. During the critical thinking curriculum pilot, both teachers and students expressed appreciation for the curriculum, indicating that it was meaningful and interactive. The training aimed at identifying critical thinking elements (e.g., analyzing arguments in readings), standards of scientific thinking (e.g., examining data for accuracy and precision), fallacies and cognitive errors (e.g., inquiring about availability bias), distinguishing fact versus opinion, and principles of scientific reasoning and presentation (Supplement 1).

### Study size

A sample size of 53 students was calculated to represent the finite population of 91 medical students with 1 study group, a continuous primary endpoint, an anticipated mean and standard deviation of the known population of 12.48±3.23 [9], a 10% buffer against drop-out in the study group, an alpha value of 0.05, and test power of 80% using the clincalc.com online sample size calculator.

### Statistical methods

Descriptive statistics were used to describe the pre- and post-test scores. The Shapiro-Wilk test of normality was performed for all the variables. Since all variables did not follow a normal distribution, we used nonparametric tests for data analysis. The Wilcoxon signed rank test was performed to compare the mean scores of the pre- and post-tests in different subscales, and the Mann-Whitney nonparametric test was used to compare the mean differences in the pre- and post-test results between male and female participants. All statistical analyses were performed using IBM SPSS ver. 20.0 (IBM Corp., Armonk, NY, USA).

## Results

We assessed the critical thinking skills of 56 medical students who completed the pre- and post-tests. Raw response data is available from Dataset 1. The CCTST (form B) was administered before and after critical thinking education during a 4-semester period. The highest score in both the pre- and post-tests was for deductive skills, while the lowest mark at both time points was for analysis (Table 1). Students’ progress was calculated by subtracting the pre-test score from the post-test score. Table 1 presents the changes in the scales. Significant changes were found in the analysis (P=0.03), evaluation (P=0.04), and inductive reasoning (P<0.0001) subscales, but not in the inference (P=0.28) and deductive reasoning (P=0.42) subscales. No significant differences were found between male and female participants (P=0.77). The results are presented in Table 2.

## Discussion

### Key results

In this study, medical students' critical thinking skills were evaluated as they progressed through the preclinical phase of the undergraduate medical curriculum. Overall, our results showed that the cohort achieved significantly higher total critical thinking scores from entry to the third year of the preclinical phase. A comparison of mean the pre- and post-test reflection scores showed that the analysis, evaluation and inductive reasoning scores improved through the curriculum integrating critical thinking. No significant difference in the pre- or post-test scores was found based on gender.

### Interpretation

The results of this study suggest that medical students have a meaningful ability to acquire critical thinking skills and that their critical thinking skills improve after delivery of direct instruction, despite relatively low scores in some critical thinking subscales. After students participated in the program, their total scores for analysis, evaluation, and inductive reasoning on the CCTST (form B) significantly improved, to a greater extent than their inference and deductive reasoning scores. Possible explanations for the statistically significant improvement include the length of the program and the explicit teaching of critical thinking. The mean scores on the pre- and post-tests of the inference and deductive reasoning subscales were lower than those of the other subscales, which may relate to the application of subscales of critical thinking skills in preclinical medical students and how they learn. Another possible explanation for this is that the medical education program at TUMS emphasized students’ capacity to analyze and engage with arguments. In other words, aspects of our program may not have supported growth in students’ hypothesis-testing and inference skills.

### Comparison with previous relevant studies

Medical students’ critical thinking skills were enhanced dramatically in all subscales, except for inference and deductive reasoning. In our study, the highest scores were seen for the analysis, inductive reasoning, and evaluation subscales. Smith et al. [10] found a significant improvement in analysis skills, while scores for the induction subscale sharply decreased. Furthermore, the findings of the current study do not support those of other previous studies. Jacob [11] reported that, after using online discussion forums, critical thinking skills showed the most noticeable improvements in the inference and deduction subscales. Chen et al. [12] reported that despite a significant increase in the critical thinking score in the intervention group, only the inference domain showed a significantly higher adjusted mean score. Although these disparities in results can be partially explained by differences among studies in terms of the context, and participants, it may be interpreted that our program and environmental factors can contribute to a consequence that does not support growth of these skills. However, another possible explanation for this is that the medical education program at TUMS emphasized students’ capacity to analyze and engage with arguments. In other words, aspects of our program may not have supported growth in students’ hypothesis-testing and inference skills. In this study, no significant differences were found between the scores of male and female students. A similar study was performed at Isfahan University of Medical Sciences during 2008–2010, using the same questionnaire to assess the critical thinking skills of medical sciences students for 2 sequential semesters, and found some similar results, including the lack of a difference between genders in total scores and relatively high scores for deductive skills [13]. However, they reported no significant improvements, and in that sense, the findings of the study are quite dissimilar to those of the present study [13]. The findings of the current study are consistent with those of Aziz-Fini et al. [14] and Liu et al. [15], who found no significant relationship between nursing students’ score in critical thinking skills and their age and gender.

### Limitations

There are some limitations of this study. Only pre- and post-test scores were included, which is inadequate for a full assessment of the longitudinal effect of the intervention. Furthermore, there was no comparison group in this study, which would have been necessary to determine whether the curriculum integrating critical thinking made a difference. In addition, the students knew that completing the questionnaires had no effect on their academic performance, which might have affected the results of the study. Further studies with different teaching strategies, larger sample sizes, and longer follow-up periods may help to achieve a better method to improve critical thinking skills.

### Conclusion

Despite its limitations, the present study makes a meaningful contribution by indicating that teaching clinical thinking to undergraduate medical students could improve their critical thinking skills, especially in terms of analysis, inductive, and evaluation skills. However, weaker performance was found for inference and deductive skills. There was no significant difference in critical thinking scores according to gender. Integrating education on critical thinking more widely into pre-clinical undergraduate medical education could enhance the shift towards scientific thinking and reasoning among medical students.

## Figures and Tables

**Table 1.** Pre- and post-test scores of the California Critical Thinking Skills Test from 56 medical students in Iran after the 2 and half-year curriculum integrating critical thinking

Critical thinking skills	Range of possible scores	Mean± standard deviation		Change	P-value
		Pre	Post		
Total score	0–34	16.09±3.23	17.41±3.87	1.32 (0.25–2.40)	0.03
Analysis	0–9	4.43±1.44	5.02±1.26	0.59 (0.06–1.12)	0.03
Evaluation	0–14	5.46±1.60	6.11±1.93	0.65 (0.06–1.22)	0.04
Inference	0–11	6.20±1.76	6.50±1.83	0.30 (0.23–0.84)	0.287
Deductive reasoning	0–16	8.36±2.08	8.62±2.11	0.26 (0.37–0.90)	0.421
Inductive reasoning	0–14	5.96±1.60	8.16±2.25	2.2 (1.53–2.86)	0.0001

**Table 2.** Changes in participants’ critical thinking scores according to gender from 56 medical students in Iran after the 2 and half-year curriculum integrating critical thinking

Critical thinking skills	Mean±standard deviation	P-value
Total scores		0.90
Male	1.15±3.36	
Female	1.47±5.56	
Analysis		0.80
Male	0.42±1.74	
Female	0.73±2.18	
Evaluation		0.90
Male	0.65±1.83	
Female	0.63±2.44	
Inference		0.22
Male	0.08±2.05	
Female	0.63±1.90	
Deductive reasoning		0.57
Male	0.35±2.44	
Female	0.20±2.38	
Inductive reasoning		0.81
Male	2.35±2.27	
Female	2.07±2.67	

## Footnotes

**Data availability**
Data files are available from Harvard Dataverse: https://doi.org/10.7910/DVN/9DZWRL Dataset 1. Response data from 56 medical students.

## Supplementary materials

Data files are available from Harvard Dataverse: https://doi.org/10.7910/DVN/9DZWRL

Supplement 1. Details of the intervention program.

Supplement 2. Survey questionnaire in Persian.

Supplement 3. Audio recording of the abstract.
