# Supplementary material for: Effects of a curriculum integrating critical thinking on medical students’ critical thinking ability in Iran: a quasi-experimental study
Source: J Educ Eval Health Prof. 2021 Jul 5;18:14. doi: 10.3352/jeehp.2021.18.14 (PMC8441095; doi:10.3352/jeehp.2021.18.14)
Supplement: Supplementary file 2 — Supplement 1. Details of the intervention program. [file jeehp-18-14-suppl1.docx]

**Supplement 1. Details of the intervention program**

**The contents of the program**

The educational program of critical thinking was arranged as a spiral, with students studying the thinking skills during the basic sciences phase of undergraduate medical education. Based on the literature review and opinion of experts, education of four areas including Components and standards of thinking (semester 1), Different types of cognitive biases and fallacies (semester 2), Fact vs. Opinion and Rhetoric (semester 3) and Principles of scientific reasoning and presentation (semester 4) was conducted (Table 1). In total, the program consisted of 21 hands on critical thinking skills lessons geared toward medical students in basic sciences phase. Educational material including pre-reading texts, worksheets, California Critical Assessment Test (form B) were prepared.

**Aim and objective of the program:**

In general, the aim of the program was promoting the students’ ability to use skillful and critical thinking in a wide range of problems which were faced. The goals of the program are as follows:

1. Explain the intellectual components.

2. Explain the standards of thinking.

3. Explain the principles of reasoning.

4. Evaluate each intellectual component based on thinking standards.

5. Apply the principles of self-assessment and correction.

6. Analysis the general principles of scientific thinking

7. Distinguish between different types of arguments (induction, analogy, etc.).

8. Discriminate the fallacies and cognitive biases in text, speech, etc.

9. Make a logical reasoning by considering the cognitive bias and fallacies.

10. Distinguish between their opinions, facts and rhetoric.

11. Analysis the process of thinking and problem solving in various fields (experimental sciences, ethics, logic and etc.)

12. Evaluate related intellectual components based on the standards of thinking and observing the principles of reasoning.

**Teaching methods of the program:**

Prior to the sessions, pre-reading texts related to the topics of the session were available for the students through the website. At the beginning of the sessions, the instructor presented the contents using a mini interactive lecture as well as question and answer technique. Subsequently, depending on the objective of each session, students responded to the worksheets individually or in small groups of six to seven people. Facilitators responded to students’ questions as well as conducting group discussions, in the process of solving the worksheets. At the end of each session, instructor summarized the golden points and feedbacks from the worksheet responses, for the students.

**Table 1: Details of content and hours of critical thinking program**

| Number of hours | Contents | Title of sessions | Semester |
| --- | --- | --- | --- |
| 14 | Structure of reasoning, Assumption, Types of claim, , Induction, Clarity, Precision, Accuracy, Relevancy, Sufficiency | Components and standards of thinking | First |
| 14 | Representative bias, Availability bias, Regression to the mean, Anchoring adjustment bias, Argument by force, Argument from pity, Guilt trap, Argument from popularity, Relativism, The ad hominem fallacy, Genetic fallacy, Straw man, False dilemma, Line-drawing | Different types of cognitive biases and fallacies | Second |
| 10 | Selective observation, Suppressed evidence, Conflict of interest, Types of supporting material, Analogy | Fact vs. Opinion and Rhetoric | Third |
| 8 | Argument components in science, Rationalization, The Impact of communications, Scientific structure of presentation, Scientific presentation contents | Principles of scientific reasoning and presentation | Fourth |
