# Supplementary material for: Effects of a curriculum integrating critical thinking on medical students’ critical thinking ability in Iran: a quasi-experimental study
Source: J Educ Eval Health Prof. 2021 Jul 5;18:14. doi: 10.3352/jeehp.2021.18.14 (PMC8441095; doi:10.3352/jeehp.2021.18.14)
Supplement: Supplementary file 3 — Supplement 2. Survey questionnaire in Persian. [file jeehp-18-14-suppl2.pdf]

آزمون ارزیابی تفکر نقاد (کالیفرنیا فرم ب)

چهار رقم آخر شماره دانشجویی..............................

۱. عبارت " علی نگران نباش، تو بالاخره یک روز در شغلت ارتقا می‌یابی تو برای یک شرکت خوب کار می‌کنی و هر کسی که برای یک شرکت خوب کار می‌کند، دیر یا زود در شغلش ارتقا پیدا می‌کند " با فرض پذیرش اینکه تمام عبارات ذکر شده صحیح است نتیجه‌گیری :

۱) صحیح است

۲) احتمالاً صحیح است اما می‌تواند غلط باشد

۳) احتمالاً غلط است و می‌تواند صحیح باشد

۴) غلط است

۲. عبارت " به آن ماشین‌ها نگاه کنید، همگی با سرعت دقیقاً در یک خط پشت سر هم در حال حرکت هستند، آن‌ها آنقدر به هم نزدیک هستند که اگر یکی از آنها توقف کند ، اتومبیل پشت سر به آنها برخورد خواهد کرد. بنابراین اگر ماشین اول ناگهان توقف کند، همه ماشین‌ها به یکدیگر برخورد خواهند کرد" با فرض اینکه تمام مطالب فوق صحیح باشد، پیش‌بینی اصلی این مطلب :

۱) صحیح است

۲) احتمالا صحیح است اما می‌تواند غلط باشد

۳) احتمالا غلط است اما می‌تواند صحیح باشد

۴) غلط است

۳. عبارت " پروژه خط لوله نفت مانند چاقویی که می تواند سیب را به دو بخش تقسیم کند شهر ما را به دو قسمت شرق و غرب تقسیم کرده است، به همین دلیل ساکنین این دو بخش بعد از این پروژه همدیگر را به عنوان اهالی یک شهر نمی شناسند . این جدایی جغرافیایی منجر به عدم اعتماد ، ترس و خصومت آشکار شده است . به همین دلیل من به این نتیجه رسیده ام که پروژه خط لوله نفت اشتباه بزرگی برای شهر ما بود" با فرض اینکه تمام مطالب فوق صحیح باشد ، نتیجه گیری گوینده:

۱) صحیح است

۲) احتمالا صحیح است اما می تواند غلط باشد

۳) احتمالا غلط است اما می تواند صحیح باشد

۴) غلط است

۴. این عبارت را در نظر بگیرید : حتی مسئولین کشور هم گاهی دچار دو دلی و شک می شوند دلیل آن این است که هر فردی که به دنبال تغییرات اساسی در ساختار جامعه باشد ، مجبور است زندگی و سرنوشت بسیاری از افراد را در خطر اندازد. مهاتما گاندی هم که به عنوان مصلح دلسوز و حامی عدم خشونت شناخته شده است به دنبال تغییرات اساسی در ساختار اجتماعی بود و هر کسی که زندگی و سرنوشت افراد را به خدا می اندازد حد اقل بعضی اوقات دچار شک و دو دلی می شود . با در نظر گرفتن اینکه تمامی دلایل ذکر شده صحیح باشد ، عبارت اول :

۱) صحیح است
۲) احتمالاً صحیح است اما می تواند غلط باشد
۳) احتمالاً غلط است اما می تواند صحیح باشد
۴) غلط است

۵. کدام یک از گزینه های زیر با عبارت زیر هم معنی است؟ " تمام مدیران برای کنفرانس حاضر نیستند" :
۱) تمام مدیران برای کنفرانس حاضر نیستند
۲) هیچ یک از مدیران برای کنفرانس حاضر نیستند
۳) کسی که برای کنفرانس حاضر است ، مدیر نیست
۴) تعدادی از مدیران ، برای کنفرانس حاضر نیستند

۶. فرض کنید این جمله صحیح باشد " فقط افرادی که به دنبال فعالیت و هیجان هستند باید به نیروی دریایی ملحق شوند " کدام یک از گزینه های زیر همین معنی را بیان می کنند؟
۱) شما نباید در جست و جوی فعالیت و هیجان باشید ، مگر اینکه به نیروی دریایی ملحق شوید
۲) شما نباید به نیروی دریایی ملحق شوید ، مگر این که به دنبال فعالیت و هیجان باشید
۳) اگر شما در جست و جوی فعالیت و هیجان هستید باید به نیروی دریایی ملحق شوید
۴) اگر شما به نیروی دریایی ملحق شوی ، باید به دنبال فعالیت و هیجان باشید

۷. فرض کنید یک زیست شناس در مورد حیوانات خانگی در حال سخنرانی است. او می گوید " سگ خلق وخوی های متعددی را نشان می دهد" کدام یک از گزینه های زیر بهترین تفسیر برای این عبارت زیست شناس می باشد؟
۱) سگی وجود دارد که بیش از یک خلق و خو دارد
۲) تمام سگ ها ، خلق و خو های متعددی دارند
۳) تمام سگ ها خلق و خو های یکسانی ندارند
۴) یک موجود وجود دارد که بیش از یک خلق و خو دارد و آن سگ است
۵) تمام تفسیر های بالا به یک اندازه قابل پذیرش هستند

۸. " مهاجر ها دردسر درست می کنند" کدام یک از گزینه های زیر همین معنی را می دهند ؟
۱) مردم دردسر درست نمی کنند ، مگر اینکه مهاجر باشند
۲) اگر فردی مهاجر باشد ، پس او دردسر درست می کند
۳) اگر فردی دردسر درست کند ، پس آن فرد مهاجر است
۴) حداقل یک فرد وجود دارد که دردسر درست می کند
۵) تمام موارد فوق یک معنی را می دهد

۹. کدام یک از گزینه های زیر تقریبا معادل این جمله است؟ " صحیح نیست که بگوییم اگر علی ماشین را تعمیر کرده ، پس رضا قایق را تعمیر کرده است"
۱) علی ماشین را تعمیر کرده ولی رضا قایق را تعمیر نکرده است

۲) علی ماشین را تعمیر نکرده مگر آنکه رضا قایق را تعمیر کرده باشد
۳) یا علی ماشین را تعمیر کرده یا رضا قایق را تعمیر کرده است
۴) اگر رضا قایق را تعمیر نکرده باشد ،پس علی هم ماشین را تعمیر نکرده است
۵) هیچ کدام از گزینه های بالا تقریبا هم معنی با عبارت ذکر شده در سوال نیستند

۱۰. این متن را در نظر بگیرید : ۱) در اکثر کشورهای صنعتی ، نوجوانان تا بعد از سن بیست سالگی به نیروی کاری جامعه نمی پیوندند. ۲) در واقع بعضی از جامعه شناسان معتقدند که پیشرفت اقتصادی یک جامعه را می توان بر اساس میانگین سن پیوستگی به نیروی کار جامعه سنجید. ۳) مطالعات روانشناسی بیان می کند که اضطراب های مختلف دوره نوجوانی در کشورهای صنعتی بیشتر آشکار هستند. ۴) به هر حال اگر فکر کنیم نوجوانانی که کار می کنند احتمال دارد که لذت و خوشحالی در محیط کار را کمتر تجربه کنند، اشتباه است.
متن بالا در جهت نشان دادن کدام یک از گزینه های زیر بهتر توصیف شده است؟
۱) اثبات درستی جمله (۱)
۲) اثبات درستی جمله (۲)
۳) اثبات درستی جمله (۳)
۴) اثبات درستی جمله (۴)
۵) هیچ کدام

جهت پاسخگویی به سوالات ۱۱ و ۱۲ متن زیر را بخوانید:

۱) جهت درستی یا نادرستی یک عمل ما باید اصول اخلاقی را بدون توجه به نتایج و پیامدهای احتمالی آن در نظر بگیریم ۲)اعمال صحیح آنهایی هستند که دارای نیت خیر، بیان حقیقت و احترام به حقوق دیگران باشند و اعمال ناصحیح آنهایی هستند که این اصول را نقض می کنند. ۳) موقعیتی را در نظر بگیرید که بیان حقیقت ممکن است منجر به زبان بزرگی برای ملت شود. برای مثال ۴) فرض کنید که شما می دانید که یک کاندیدای ریاست جمهوری سالها پیش دارای سابقه فساد اخلاقی بوده است. ۵) فرض کنید شما می دانید که اگر این کاندیدا انتخاب شود، مشکلات خارجی و داخلی کشور مطمئناً حل خواهد شد، غرور ملی احیا می گردد و نام او به عنوان یکی از بزرگترین رئیس جمهورها در تاریخ ثبت خواهد گردید. ۶) اما شما می دانید که اگر عموم مردم از سابقه فساد اخلاقی گذشته او آگاهی یابند او مطمئناً تمام شانسها را برای انتخاب شدن به عنوان رئیس جمهور از دست خواهد داد. ۷) با این حال وقتی که از طرف رسانه ها سوالات اختصاصی در مورد سابقه فساد اخلاقی این کاندیدا پرسیده می شود شما نمی توانید از پاسخ دادن اجتناب کنید. ۸) بیان حقیقت مستلزم آشکار کردن سابقه فساد اخلاقی گذشته او می باشد. ۹) بنابراین بیان حقیقت می تواند عمل درستی باشد حتی اگر منجر به ضرر و زیان بزرگی برای کل ملت ما باشد.

۱۱. کدام یک از جملات متن بالا بیانگر نتیجه گیری اصلی است؟
۱) جمله ۱
۲) جمله ۲
۳) جمله ۳

۴) جمله ۸

۵) جمله ۹

۱۲. بهترین توصیف برای جمله ۲ در متن بالا کدام یک از گزینه های زیر است؟

۱) یک جمله ربطی که جملات ۱ و ۳ را به هم ربط می دهد.

۲) یک نقطه نظر اخلاقی که از نظر منطقی بی ارتباط (نا مرتبط) می باشد.

۳) دلیلی جهت تایید جمله اول

۴) نتیجه گیری یا نقطه نظر اصلی متن

۵) توضیح و یا رفع ابهامی جهت جمله اول

۱۳. اخیراً بسیاری از سازمانها بر مبنای تعاونی ایجاد شده اند این امر نشان دهنده این مطلب است که در رویکردهای پیشرفته تر جهت دستیابی به بازار، تعاونی مورد توجه و علاقه قرار گرفته است. این متن توسط کدام یک از گزینه های زیر بهتر توصیف می شود؟

۱) نتیجه گیری " مدیریت خواستار راهکارهای جدید جهت دستیابی به بازار می باشد".

۲) نتیجه گیری " تعاونی ها اساساً وجود داشته اند و منحصراً جهت تامین منابع صاحبانشان نبوده اند".

۳) نتیجه گیری " تعاونی ها به زودی بهترین عمل جهت رسیدن به بازار خواهند شد".

۴) پیش فرض" تعاونی ها قبل از توسعه سازمانها، جهت رسیدن به بازار ناموفق بوده اند".

۵) پیش فرض " سازمان های جدید یاد شده جهت رسیدن به بازار با توجه به رویکردهای جدید و پیشرفته در حال فعالیت می باشند".

۱۴. این عبارت را در نظر بگیرید: " جولیوس سزار" در قرن اول پیش از میلاد مسیح امپراطور روم بود. تمام امپراطوران روم شراب می نوشیدند و بدون استثناء از ظروف و جام هایی که دارای الیاژ سرب بود استفاده می کردند هرکسی که از ظرف سربی استفاده کنند، حتی برای یکبار، مسموم خواهد شد و مسمومیت با سرب همیشه خودش را با جنون نشان می دهد".

اگر تمام موارد فوق صحیح باشد کدام یک از گزینه های زیر باید صحیح باشند؟

۱) مسمومیت با سرب در بین شهروندان امپراطوری روم رایج بوده است.

۲) استفاده از ظرف سربی مزیتی منحصر به امپراطوران روم بوده است.

۳) صرف نظر از هر امری، جولیوس سزار مطمئناً دیواه بوده است.

۴) کسانی که جنون دارند حداقل یکبار از ظروف سربی استفاده کرده اند.

۱۵. فرض کنید این عبارات صحیح باشند: " افرادی که شیک‌پوش و اهل مد روز هستند نه خودنما هستند و نهکودن، اگر چنین شخصی خودنما نیست پس چنین فردی خوش سلیقه است". در صورتی که مطالب ذکر شده صحیح باشند کدام یک از گزینهای زیر باید صحیح باشد؟

۱) اگر فردی لباس شیک بپوشد، آن فرد کودن اما خوش سلیقه است.

۲) هیچ فرد خوش سلیقه ای کودن نیست.

۳) افرادی که لباس شیک می پوشند نه خوش سلیقه هستند نه کودن.

۴) هر فردی که لباس شیک می پوشد خوش سلیقه است اما کودن نیست.

۵) هیچ یک از موارد فوق

۱۶. فرض کنید عبارات زیر صحیح باشند:" اگر علی به شخصی حسادت بورزد، آن فرد رضا است. افراد زیادی هستند که رضا به آنها حسادت نمی کند که علی یکی از آنهاست اما در دنیای امروزه هر فردی به دیگری حسد می ورزد" اگر تمام مطالب فوق صحیح باشد، کدام یک از گزینه های زیر باید صحیح باشد؟

۱) هر فردی به یک نفر حسد می ورزد.

۲) علی به رضا حسد می ورزد.

۳) رضا به هیچ کس حسد نمی ورزد.

۴) هیچ کدام از موارد فوق

سوالات ۱۷ و ۱۸ بر اساس موقعیت تخیلی زیر می باشند:

شهر مشهد دارای هفت منطقه شامل منطقه ۷، ۶، ۵، ۴، ۳، ۲، ۱ می باشد. شهردار باید جهت عضویت در شورای شهر، نام پنج نفر را که هر کدام از یک منطقه هستند اعلام نماید. هر نوع ترکیبی از ۵ نفر امکان پذیراست جز اینکه، در صورت اعلام نام فردی از منطقه ۱، نمی توان عضوی را از منطقه ۵ برگزید اما اگر فردی از منطقه ۳ انتخاب شود، انتخاب عضوی از منطقه ۵ الزامی ست و هم‌چنین اگر فردی از منطقه ۲ انتخاب شود، شهردار باید عضوی را از منطقه ۶ نیز اعلام نماید.

۱۷. از بین ۵ ترکیب احتمالی که شهردار می تواند جهت انتخاب اعضای شورای شهر داشته باشد کدام یک تمامی موارد فوق را دارا می باشند؟

۱) ۷، ۶، ۳، ۲، ۱

۲) ۷، ۶، ۵، ۴، ۱

۳) ۷، ۶، ۴، ۳، ۲

۴) ۶، ۵، ۴، ۳، ۲

۵) ۶، ۵، ۴، ۲، ۱

۱۸. فرض کنید که شهردار تصمیم دارد که فردی را از منطقه ۷ انتخاب نکند، در این صورت کدام منطقه دیگر باید از عرصه انتخاب شورای شهر حذف شود؟

۱) منطقه ۱

۲) منطقه ۲

۳) منطقه ۳

۴) منطقه ۴

۵) منطقه ۵

۱۹. رابطه " همزاد بودن" را در نظر بگیرید. آن را می توان این چنین تعریف: " فقط انسانها همزاد هستند. اما هر فردی از گونه انسانها همزاد ندارد. هیچ فردی نمی تواند همزاد خود باشد، اما امروزه فردی همزاد فرد دیگری است" اگر فردی همزاد شما

باشد، پس تمام همزاد های آن فرد، همزاد شما هم خواهند بوداگر فردی همزاد شما باشد، پس شما نمی توانید همزاد همان فرد باشید. فرض کنید نام دو انسان اولیه که ما نیاکان ما به شمار می آیند؛ آدم و حوا باشد. با در نظر گرفتن مسئله همزادی با اطمینان می توان گفت :

1) تمام ما همزاد آدم و حوا هستیم.
2) آدم و حوا همزاد یکدیگر هستند.
3) آدم و حوا هر یک همزاد خودشان هستند.
4) امکان دارد که فردی نه همزاد آدم و نه همزاد حوا باشد.
5) هیچ کدام از موارد فوق

برای پاسخ گویی به سوالات ۲۰ و ۲۱ از موقعیت تخیلی زیر استفاده کنید:

در یک مطالعه علمی از زنان دانشگاهی که حداقل به مدت ۲ سال روزانه یک یا چندین سیگار کشیده بودند ۴۵ روز پس از ترک سیگار با انجام آزمایشی مشاهده شد که حجم ریوی ۸۵ درصد از آنها، ۱۵ درصد افزایش یافت این احتمال که بهبودی مزبور تصادفی و یا از روی شانس باشد، به صورت تجربی با درجه بالایی از اعتماد رد شد.

۲۰. اگر عبارات فوق صحیح باشد، یافته های فوق اثبات می کند که:

1) سیگار کشیدن سبب کاهش ظرفیت ریوی می شود.
2) محدودیت استعمال دخانیات در مراکز دانشگاهی باید به اجرا درآید.
3) رژیم غذایی یک عامل در رابطه بین سیگار کشیدن و ظرفیت ریوی نیست.
4) پژوهشگر علاقه‌ای ذاتی به ترک مصرف دخانیات دارد.
5) مصرف سیگار از نظر آماری با کاهش ظرفیت ریوی در زنان دانشگاهی رابطه دارد.

۲۱. اگر اطلاعات ذکر شده در موقعیت بالا صحیح باشد، کدام یک از فرضیات زیر، به منظور اثبات این ادعاکه " حدود ۸۵ نفر از ۱۰۰ نفر بزرگسالی که حداقل به مدت ۲ سال روزانه یک یا چندین پاکت سیگار می کشیده‌اند در طی ۴۵ روز پس از ترک سیگار می توانند ۱۵ درصد ظرفیت ریوی خود را بهبود ببخشد" نباید رد شود؟

1) بهبود ظرفیت ریوی محدود به زنان است اما در مردانی که سیگار را ترک کرده‌اند این بهبود بارز نخواهد بود.
2) به علت اینکه افراد سیگاری، تعداد مصرف سیگار خود را گزارش نموده‌اند رابطه واقعی بین ترک سیگار و بهبود ظرفیت ریوی بیش از میزان ذکر شده می باشد.
3) از آن جا که زنان مورد مطالعه اکثراً آسیایی بوده‌اندیافته‌های مزبور را نمی توان برای افراد بزرگسال جوامع دیگر به طور عمومی بکار برد.
4) از آنجا که مسئولین دانشکده نتوانستند موضوع تحقیق را محرمانه نگه دارندزنان دانشگاهی و دانشمندان دست اندرکار از هدف مطالعه اطلاع داشتند.

۲۲. فرض کنید که هر موقع که قطار تاخیر می کند، علی و رضا گرسنه و بی قرار می شوند با در نظر گرفتن این فرض کدام یک از گزینه های زیر باید صحیح باشد؟

۱) قطار تاخیر دارد، علی گرسنه و رضا بی قرار است.

۲) اگر علی گرسنه باشد اما رضا بی قرار نباشد، قطار تاخیر ندارد.

۳) اگر علی بی قرار و یا رضا گرسنه باشد، قطار تاخیر دارد.

۴) اگر قطار تاخیر نکند، علی و رضا نه بی قرار هستند نه گرسنه.

۵) اگر علی و رضا گرسنه یا بی قرار باشند، قطار تاخیر دارد.

۲۳. یک مدیر حسابداری هنگام کار کردن بر روی یک مشکل با ارزیابی چنین استدلال می کند که " طرح الف" از "طرح ب" بهتر می باشد. با این حال " طرح م" از " طرح د" بهتر است. بنابراین " طرح د" از " طرح ن" بهتر می باشد. با فرض درستی تمام موارد فوق کدام یک از گزینه های زیر باید به بحث حسابدار اضافه شود تا نتیجه گیری نهایی درست باشد؟

۱) " طرح ن" بدتر از " طرح د" است.

۲) " طرح ن" بدتر از "طرح الف " است.

۳) "طرح ب" بدتر از " طرح ن " است.

۴) "طرح الف" بدتر از "طرح ن" است.

۵) هیچکدام از موارد فوق

جهت پاسخ گویی به سوالات ۲۴ و ۲۵ موقعیت تخیلی زیر را در نظر بگیرید:

نتایج پژوهش در ۱۵ دانشگاه دولتی در ارتباط با " آزمون استاندارد سنجش آمادگی عمومی شغلی" نتایج زیر را نشان داد: میانگین نمرات دانشجویان ترم آخر رشته های علوم پایه، مهندسی و تجارت به ترتیب ۵۳ و ۵۵ و ۵۴ بود. مطالعه دیگری که بر روی دانشجویان ۱۰ دانشگاه خصوصی انجام شده بود نشان داد که میانگین نمرات دانشجویان در همان آزمون در رشته های علوم پایه، مهندسی و تجارت، ۵۶ اما در رشته علوم انسانی ۵۴ بود. مطالعه سوم بر روی یک گروه انتخاب شده از جوانانی که در دوره دبیرستان دانش آموزان خوبی بوند و فقط به دلیل مشکلات مالی به دانشگاه راه نیافته بودند و پس از اتمام دوره دبیرستان مستقیماً به کار تمام وقت مشغول بودند، انجام شد. این گروه از نظر سن و معلومات دوره دبیرستان با دو گروه دیگر همگون شده بودند میانگین نمره این افراد در همان آزمون ۳۲ بود که اختلاف عدد۳۲ با نمرات فوق از نظر آماری با ۰/۰۵ اعتماد معنی دار بود.

۲۴. مناسب ترین فرضیه علمی بر اساس عبارات فوق عبارت است از:

۱) تحصیلات دانشگاهی با آمادگی عمومی شغلی رابطه دارد.

۲) باید برای دانش آموزان مستعد و کم بضاعت جهت ورود به دانشگاه کمک های مالی فراهم گردد.

۳) ورود به دانشگاه با آمادگی عمومی جهت ورود به یک شغل رابطه ندارد.

۴) قبل از تدوین یک فرضیه مناسب، مطالعات بیشتری نیاز است.

۵) فردی که نمره ۶۰ یا بالاتر می گیرد، عموماً آمادگی لازم جهت وارد شدن به یک شغل را دارد.

۲۵. جهت رد علمی گزینه ۳ در سوال ۲۴ فرد باید:

۱) دانشجوی فارغ التحصیلی را بیابد که آمادگی ورود به شغل را ندارد.

۲) به این نتیجه برسد که ۹۵ درصد جوانان به طور عمومی آمادگی ورود به شغل را دارند.

۳) به این نتیجه برسد که احتمال ایجاد رابطه تصادفی کمتر از ۵ در صد می باشد.
۴) دست به هیچ اقدامی نزند، هیچ راهی جهت رد علمی نظریه مزبور وجود ندارد.

۲۶. به نظر می رسد دو دلیل اصلی برای طرفداری از مجازات اعدام وجود دارد. دلیل اول اینکه ترس شدید ناشی از مرگ دیگران را از ارتکاب جنایات وحشتناک مشابه، باز می دارد و دلیل دوم این که اعدام اقتصادی تر از روش دیگر یعنی زندگی کردن در زندان می باشد. اما، مطالعات علمی نشان داده است که واقعیات اقتصادی شدیداً طرفدار زندانی کردن افراد می باشند. مردم عموماً فکر می کنند که صرفه جویی اقتصادی در مجازات اعدام واقعیت های اقتصادی را تغییر نمی دهد. بنابراین مجازات اعدام باید ممنوع گردد. دلایل ذکر شده گوینده عبارات فوق را چگونه ارزشیابی می کنید؟

۱) ضعیف نشان دهنده نظرات عموم مردم نیست.
۲) ضعیف این استدلال دلیلی در ارتباط با منع دیگران از ارتکاب جنایات ارائه نمی دهد.
۳) قوی، این استدلال نشان می دهد که مجازات اعدام احتمالاً باید ممنوع گردد.
۴) قوی، اما در ارتباط با ممنوعیت مجازات اعدام به طور واقعی اشتباه کرده است.

۲۷. متوسط قیمت فروش خانه در کشور در طی یک دوره به شدت کاهش یافت و این روند در طول این دوره که سه سال به طول انجامید ادامه داشت. در طول همین دوران نرخ بهره و قیمت املاک شدیداً افت نمود. این واقعیات نشان دهنده این امر است که خانه جزو اکلاک به شمار می آید. بهترین ارزشیابی از دلایل گوینده عبارت است از:

۱) قوی ، اما تمام واقعیتها به طور صحیح بیان نشده است.
۲) قوی، زیرا خانه جزو املاک به شمار می آید.
۳) ضعیف، زیرا با در نظر گرفتن واقیات مربوط به املاک و نرخ بهره نمی توان در مورد قیمت خانه نتیجه گیری کرد.
۴) ضعیف،زیرا قیمت فروش اتومبیل های جدید در طی همان دوران کاهش یافته بود اما این مسئله نشان دهنده این مطلب نیست که خانه، همان اتومبیل جدید است.

۲۸. همزمان با غروب آفتاب، علی کوچولو در پیاده روی جلوی منزل دوچرخه سواری می کرد. شب در حال فرا رسیدن بود و مهمانی شبانه او قرار بود شروع شود. علی تمام دوستانش را دعوت کرده بود. آنها قرار بود پیتزا بخورند، فیلم های جالب ببینند و تا دیروقت داستانهای ترسناک برای یکدیگر تعریف کنند علی بی صبرانه منتظر بود او آرزو کرد که خورشید سریعتر برود و پشت تپه ها قایم شود و اجازه بدهد که شب بیاید او تصمیم گرفت که با حداکثر سرعت روی دوچرخه اش پا بزند تا بتواند خورشید را دور کند. او پا زد و پا زد و هرچقدر که بیشتر پا می زد هوا تاریکتر می شد. شب در حال آمدن بود و مهمانی شبانه در حال فرارسیدن علی بیشتر و بیشتر پا زد و وقتی که سر انجام هوا تاریک شد او خیلی خسته شده بود اما خیلی خوشحال بود. علی به آنچه اتفاق افتاد اندیشید و به این نتیجه رسید که او می تواند هر بعدازظهر طولانی و خسته-کننده‌ای را به یک شب شاد تبدیل کند. بهترین ارزشیابی از دلایل علی عبارت است از:

۱) قوی، چه شاهدی وجود دارد که اگر او آنقدر سخت کار نکرده بود، آن اتفاق نمی افتاد؟
۲) قوی، علی فقط یک بچه است.
۳) ضیف، خورشید بدون ارتباط با پدال زدن یا نزدن او در حال حرکت با دور زمین است.
۴) ضعیف، اینکه بعد از پدال زدن فراوان علی آن اتفاق افتاد به این معنی نیست که بدلیل پدال زدن سخت او این اتفاق افتاد.

۲۹. گوینده رادیو بیان کرد: روزنامه نگاران باید به حقوق مردم جهت آگاهی از مطالب آشنایی داشته باشند تا بر مبنای این حق، تمام وقایع مهم را به صورت کامل و دقیق به اطلاع عموم مردم برسانند در عین حال روزنامه نگاران نقش خود را در حراست از امنیت ملی نباید فراموش کنند که لازم آن حفظ اسرار حکومتی است. هیچ کس با قطعیت نمی تواند بگوید که حق مردم جهت اطلاع از امور مختلف مهم تر است یا امنیت ملی این مساله مباحث پیچیده ای را به دنبال دارد. برای مثال یک روزنامه نگار ممکن است از زمان و محل دقیق یک حمله نظامی فوق سری تحت حمایت دولت مطلع گردد همچنین مردم حق دارند که از اقدامات دولت خصوصاً در مسایل بسیار حساس مثل حمله نظامی اطلاع داشته باشند اما انتشار این اخبار قبل از شروع حمله می تواند به نفع دشمن تمام شود و شکست سختی را برای کشور به دنبال آورد. بهترین ارزشیابی در مورد استدلال گوینده عبارت است از:

۱) ضعیف، زیرا قانون تصریح کرده که امنیت ملی مهمتر است.

۲) ضعیف، زیرا در عمل روزنامه نگاران، انتخابی را که دارای ارزش بیشتری است انجام می دهند.

۳) قوی، زیرا حقوق مردم جهت اطلاع از واقعیات را نباید زیر پا گذاشت.

۴) قوی، زیرا در متن ذکر شده ارزشهای مهم مزبور در تعارض هستند.

۳۰. مجموعه کامل ظروف غذاخوری شامل حداقل ۶ بشقاب برنج خوری، ۶ بشقاب خورش خوری، ۶ ظرف سوپخوری، ۶ لیوان و ۶ ظرف ماست خوری می باشد. می توان این ۳۰ قطعه را اساسی ترین ظروف در یک سرویس غذاخوری فرض کرد. در یک سرویس کامل غذاخوری قطعات دیگری نیز وجود دارند، از جمله کاسه های کوچک ترشی خوری، دیس های بزرگ غذاخوری، نمکدان، شکرپاش و حتی جاکره ای این قطعات را سرویس لوازم فرعی گویند. حال فرض کنید که شما یک سرویس کامل غذاخوری هدیه گرفته اید، بنابراین بر اساس آنچه گفته شد می توان نتیجه گیری کرد که: در بین قطعات این سرویس کامل ، حتماً ۶ عدد ظرف برنج خوری، خورش خوری، سوپخوری،ماست خوری و لیوان وجود دارد. بهترین ارزشیابی در مورد شیوه نتیجه گیری نویسنده عبارت است از اینکه:

۱) ضعیف، هیچ چیزی را اثبات نمی کند، زیرا مثل این است که بگوییم " اقیانوس آب است زیرا آب دارد".

۲) ضعیف، زیرا قطعات مربوط به سرویس لوازم فرعی را در نگرفته است.

۳) قوی، نویسنده انواع ظروف را در مجموعه سرویس غذاخوری برشمرده است.

۴) قوی، نتیجه گیری در واقع تکرار صریح اطلاعات فوق است.

جهت پاسخگویی به سوالات ۳۴، ۳۳، ۳۲، ۳۱ به استنتاج غلطی که در موقعیت تخیلی زیر وجود دارد توجه کنید:

نویسنده ای که با جهت گیری فکری کار می کرد مدعی شد که فارس ها از نقطه نظر هوش ذاتی بر ترکها، کرد ها، لرها، بلوچ ها و سایر اقوام ایرانی برتری دارند. نویسنده جهت تایید ادعایش به مطالعه ای که روی دو گروه از دانش آموزان سال دوم دبیرستان انجام شده بود اشاره نمود از هر گروه امتحان مشابهی در خصوص جغرافیای جهان مشتمل بر کشورها، پایتختها، رودخانه ها، رشته کوه ها، کشاورزی، صنعت، مذهب، موسیقی و زبان به عمل آمده بود. گروه اول شامل ۳۵ نفر بودند که ۳۴ نفرشان فارس و دارای خانواده مرفه بودند که در دبیرستان غیر دولتی در منطقه شمار شهر تهران مشغول به تحصیل بودند و طی سال تحصیلی علاوه بر جغرافیای ایران جغرافیای کشورهای جهان را نیز می خواندند. گروه دوم شامل ۴۰ نفر بودند که همگی به جز ۴ نفر ترک ، کرد، لر

یا بلوچ بودند و در شهرستان های ایران در خانواده های کم درآمد و در دبیرستان های دولتی در محله های مخروبه و پر از مفاسد اجتماعی مشغول به تحصیل بودند. و در سال اول مجموعه جغرافیای ایران را مطالعه می کردند. نویسنده اعلام نمود که گروه اول مزیت محسوسی در امتحان جغرافیا در قیاس با گروه دوم داشتند.

۳۱. فرض کنید که یک دانشمند علوم سیاسی اعتراض کند و بگوید نتیجه گیری از اطلاعات فوق با توجه به ادعای مطرح شده غلط است زیرا پژوهشگر به حقوق تصریح شده در قانون اساسی مبنی بر برخورداری از فرصتهای آموزشی یکسان توجه ننموده است. در صورت درست بودن این مطلب آیا دلیل دانشمند موجه است یا خیر چرا؟

۱) موجه، زیرا زیر پا گذاشتن اصول قانون اساسی ، مطالعه را غیر قابل قبول می سازد.

۲) موجه، وجود فرصتهای آموزشی یکسان در اینجا گنگ و نامفهوم است.

۳) غیر موجه، این حقوق در تحقیق اصلی مورد توجه قرار گرفته اند.

۴) غیر موجه، این حقوق هیچ ارتباطی با تحقیق ندارد.

۳۲. فرض کنید که یک دانشمند رشد و تکامل بیان می کند: " نتیجه گیری از این اطلاعات با توجه به ادعای مطرح شده غلط می باشد زیرا در این مطالعه ، تاثیر محیط بر روی هوش مورد توجه قرار نگرفته است" در صورت درست بودن آیا دلیل دانشمند موجه است یا خیر؟ چرا؟

۱) غیر موجه، هیچ کس ثابت نکرده است که محیط می تواند بر روی یادگیری جغرافیا تاثیر بگذارد.

۲) غیر موجه، اندازه گیری تاثیر محیط بر روی هوش خیلی دشوار است .

۳) موجه، عامل محیط در این تحقیق باید مورد توجه قرار گیرد .

۴) موجه، عامل اصلی تعیین کننده هوش ژنتیک نمی باشد بلکه محیط است.

۳۳. فرض کنید که یک خانم مددکار اجتماعی اعتراض کند که : شما نباید انتظار داشته باشید که افراد گروه "ب" به همان اندازه باهوش باشند زیرا آنها دارای پیش زمینه فقر، جنایت و خانواده های از هم پاشیده هستند. در صورت درستی این مطلب آیا دلیل مددکار اجتماعی است یا خیر؟ چرا؟

۱) موجه، محله فقیر به معنای مدارس فقیر، مدارس فقیر به معنای معلمان فقیر ، معلمان فقیر به معنای دانش آموزان فقیر و دانش آموزان فقیر به معنای نمرات ضعیف در آزمون می باشند.

۲) دلیل غیر موجه، صرف نظر از شرایط اجتماعی اقتصادی، هوش به کیفیت مدرسه ای که شما در آن تحصیل می کنید بستگی دارد.

۳) دلیل غیرموجه، ثروت و شرایط خانوادگی بر میزان بالا و پایین بودن هوش فرد موثر نیست.

۴) دلیل موجه، بدون توجه به اقوام مختلف، دانش آموزان با این نوع پیش زمینه ها دارای هوش کمتری نسبت به دانش آموزان ثروتمند هستند.

۳۴. فرض کنید که یک دانشجوی تربیت معلم کُرد، با عصبانیت اعتراض می کند که " شما چه انتظاری دارید؟ دانش آموزان ثروتمند درس جغرافیای ایران را گزرانده بودند. مسلماً دانش آموزان ثروتمند در مورد کشورهای مختلف جهان مطالب بیشتری می دانند." در صورت درستی این مطلب آیا دلیل او موجه است یا خیر؟ چرا؟

۱) غیر موجه، او تنها یک دانشجوی تربیت معلم است و احتمالاً تجربه تحقیق و آموزش جهت تایید ادعاهایش را ندارد.

۲) موجه، اطلاعات در مورد واقعیت ها ابزار سنجش هوش نیست.

۳) موجه، اختلاف بین دروس تدریس شده در سال اول باعث شده که گروه اول در زمینه امتحان جغرافیا از آمادگی بیشتری نسبت به گروه دوم برخوردار باشند.

۴) غیر موجه، او به شکل موضع گرفته است زیرا کُرد است و به دلیل نتایجی که نویسنده ارائه داده احساس توهین می کند.
